# Inflammation and neurological adverse drugs reactions: a case of long lasting impaired consciousness after oxatomide administration in a patient with gastroenteritis

**DOI:** 10.1186/1824-7288-38-11

**Published:** 2012-03-30

**Authors:** Stefania Antoniazzi, Dario Cattaneo, Valentina Perrone, Carla Carnovale, Simonetta Cherubini, Maria Carmela Mugolino, Francesco Clementi, Gianvincenzo Zuccotti, Emilio Clementi, Sonia Radice

**Affiliations:** 1 Unit of Clinical Pharmacology, Department of Clinical Sciences, University Hospital "Luigi Sacco", Università di Milano, 20157 Milan, Italy; 2 CNR Institute of Neuroscience, 20129 Milan, Italy; 3 Unit of Clinical Paediatric, Busto Arsizio Hospital, 20152 Busto Arsizio, Varese, Italy; 4 Department of Pediatrics University Hospital "Luigi Sacco", Università di Milano, 20157 Milan, Italy; 5 E. Medea Scientific Institute, 23842 Bosisio Parini, Lecco, Italy

**Keywords:** Oxatomide, Long lasting impaired consciousness, Adverse drug reaction, Inflammation, CYPs

## Abstract

Oxatomide at therapeutic doses generates occasionally drowsiness in children. When administered at toxic doses, however oxatomide may induce long lasting impaired consciousness. We now report a case of severe long lasting impaired consciousness induced by therapeutic doses of oxatomide occurring in a child affected by acute gastroenteritis. The clinical symptoms, the pharmacogenetic tests of polymorphisms in cytochrome P450 metabolizing enzymes (CYPs) and the clinical and laboratory analyses indicate that the enhanced drug sedative effect is likely due to an acute, yet mild, inflammatory state of the patient. These findings highlight the importance of assessing common, not serious inflammatory states when oxatomide is prescribed in paediatric patients.

## Background

Oxatomide is a H1-histamine receptor antagonist effective in treatment of allergic rhinitis, urticaria, pruritus dermatitis, eczema dermatitis, bronchial asthma and conjunctivitis. The drug inhibits the secretion of several mediators of inflammation from human basophils and mast cells [1]. Studies in neutrophils have also shown that it inhibits arachidonic acid mobilization, and the synthesis of leukotriene B4 and platelet-activating factor, possibly by reducing the activities of cytosolic phospholipase A2 [1]. Studies in human liver microsomes [2] have shown that oxatomide is mainly metabolized by cytochromes 3A4 (CYP3A4) and 2D6 (CYP2D6), two enzymes encoded by highly polymorphic genes [3,4]

Whereas at toxic dosages oxatomide may cause coma and long-lasting impaired consciousness, it is generally well tolerated at therapeutic dosage, although slight drowsiness, similarly to other H1-histamine receptor antagonists, may occur in approximately 50% of the patients [5]. To date only one study reported the occurrence of long-lasting impaired consciousness in paediatric patients at therapeutic oxatomide dosages. No pathogenic causes were described for such an effect [6].

## Case presentation

A 3 year-old Caucasian male, weighing 13.2 Kg, treated for conjunctivitis with oxatomide (TINSET^® ^- drops 2.5%) at therapeutic doses (14 mg/day for 2 days) experienced at home abdominal pains, pallor, trouble speaking followed by a serious long-lasting impaired consciousness two days after oxatomide treatment. The parents also reported muscular hypotonia. After 2 days the parents accessed the Emergency Department where the child was immediately admitted. The child had no history of recent trauma, seizures and neurologic diseases and was receiving no other drug treatment. The child was affected by acute gastroenteritis.

After admission to the Emergency Department oxatomide was withdrawn. Nonetheless, the patient's state of impaired consciusness persisted with very low response to pain stimulation tests and a Glasgow coma score of 4, which led to immediate recovery to the Paediatric Unit emergency section where all appropriate measures were taken.

The brain Computed Tomography Scan was negative with normal deep tendon reflexes. The results of blood gas analysis, serum electrolytes levels, hepatic and renal tests were normal. The cerebrospinal fluid was clear and colourless with normal protein levels and a negative cytological test. Likewise negative in the cerebrospinal fluid were all virological tests (Herpes Simplex1 and 2, Varicella Zooster and enteroviruses), and tests for mycetes, *M. tuberculosis *and Gram positive and negative bacteria. The urinalysis excluded infections by *Streptococcus pneumoniae, Haemophylus influenzae, Neisseria meningitis *and *Streptococcus agalactiae*. Moreover, the urinalysis was negative for barbiturates, benzodiazepines, opiates, cocaine, amphetamine, methamphetamine and antidepressant drugs that could have increased the sedative properties of oxatomide. The stool cultures for *Salmonella tiphymurium, Shigella, Campylobacter *were also negative. The results of haematochemical tests showed elevated leukocytes (12.77*10e3/uL) and a CRP value of 0.47 mg/dL (physiologic range < 0.25 mg/dL) while the white blood count revealed a decreased number of neutrophils (39.5%); these results are compatible with a slight clinical pictures of inflammation due to an intercurrent gastroenteritis.

The patient was hospitalized in the Paediatric Unit and forty-eight hours after oxatomide withdrawal, the patient's conditions although asthenia and headache persisted, remitting completely in the days.

Based on the clinical data the physicians excluded an acute encephalitis and correlated the symptoms to an oxatomide adverse effect. Since the patient was treated with a standard dose of oxatomide as the only therapy it was initially hypothesized that the observed over-response to oxatomide was due to a inherited defect in the activity of cytochromes CYP3A4 and/or CYP2D6, ultimately resulting in impaired drug metabolism and drug accumulation. The patient was therefore screened for the main allelic variants of CYP3A4 (CYP3A4*1B) and CYP2D6 (namely CYP2D6*3, *4, *5 and *6). However, none of the above mentioned polymorphisms were detected by pyrosequencing analysis performed using the diagnostic kits Diatech Explera according to the manufacturer's instructions.

The Naranjo adverse drug reaction probability scale [7] identified the relationship between the patient's development of ADR and the drug as "possible".

The only published article of a long-lasting impaired consciousness after therapeutic oxatomide treatment was in 1986 [6], when Van Daele *et al. *reported six cases of acute dystonic reactions and long-lasting impaired consciousness, occurring 30-60 after hours the start of treatment, in patients treated with "more or less normal" doses of oxatomide. The children showed altered consciousness varying from lethargy and somnolence to frank encephalitis.

The causes for those episodes of acute toxicity to oxatomide were not known. Our results from pharmacogenetic screening exclude for our patient inherited defects in the ability of metabolizing oxatomide. The age-dependent variability in the activity of some CYP may also be excluded. CYP3A4 activity, weak in the foetus and after birth, becomes very similar to that of an adult within the first years of life; likewise, CYP2D6 activity in infants and children up to five years of age is above two-third of the average adult levels [8]. We could not carry out a pharmacokinetic profile of oxatomide serum levels. Thus, the possibility that changes in expression of wild type CYP may have contributed to the observed ADR by oxatomide cannot be excluded.

Clinical and preclinical studies indeed show that increases in proinflammatory molecules such as interleukins (IL-1, IL-6, IL-8), tumour necrosis factor-α (TNF-α) and interferon-γ is associated with downregulation of expression and activity of several cytochromes including CYP3A4 and CYP2D6 [9]. Indeed IL-6, IL-8 and TNF-α concentrations in patients with gastroenteritis are significantly increased [10,11]. It is therefore conceivable that the release of these proinflammatory molecules has decreased CYP activities, eventually leading to increased oxatomide concentrations in the plasma of our patient, thus contributing to the observed neurological ADR.

## Conclusions

Based on our clinical observations and the data from the literature we conclude that clinicians and researchers must be extremely careful to assess common, not serious inflammatory states when prescribing oxatomide, in order to prevent serious neurological ADRs of this drug and improve safety in the paediatric clinical practice. The possibility to prescribe non sedating anti-histamine drugs for allergy should be considered.

## Consent

Written informed consent was obtained from the patients' guardians for publication of this report and any accompanying data.

## Competing interests

The authors declare that they have no competing interests.
